# Positive genetic correlation between brain size and sexual traits in male guppies artificially selected for brain size

**DOI:** 10.1111/jeb.12608

**Published:** 2015-03-11

**Authors:** A. Kotrschal, A. Corral‐Lopez, S. Zajitschek, S. Immler, A. A. Maklakov, N. Kolm

**Affiliations:** ^1^ Department of Animal EcologyEvolutionary Biology CentreUppsala UniversityUppsalaSweden; ^2^ Department of Zoology/EthologyStockholm UniversityStockholmSweden; ^3^ Department of Ecology & Genetics/Evolutionary BiologyEvolutionary Biology CentreUppsala UniversityUppsalaSweden; ^4^ Department of BiologyThe George Washington UniversityWashingtonDCUSA

**Keywords:** artificial selection, brain size, genetic correlation, guppy, sexual selection, trade‐off

## Abstract

Brain size is an energetically costly trait to develop and maintain. Investments into other costly aspects of an organism's biology may therefore place important constraints on brain size evolution. Sexual traits are often costly and could therefore be traded off against neural investment. However, brain size may itself be under sexual selection through mate choice on cognitive ability. Here, we use guppy (*Poecilia reticulata*) lines selected for large and small brain size relative to body size to investigate the relationship between brain size, a large suite of male primary and secondary sexual traits, and body condition index. We found no evidence for trade‐offs between brain size and sexual traits. Instead, larger‐brained males had higher expression of several primary and precopulatory sexual traits – they had longer genitalia, were more colourful and developed longer tails than smaller‐brained males. Larger‐brained males were also in better body condition when housed in single‐sex groups. There was no difference in post‐copulatory sexual traits between males from the large‐ and small‐brained lines. Our data do not support the hypothesis that investment into sexual traits is an important limiting factor to brain size evolution, but instead suggest that brain size and several sexual traits are positively genetically correlated.

## Introduction

Brain size is highly variable among animals, but despite over a century of research in this area, our understanding of the evolutionary processes and mechanisms that have generated this variation remains inconclusive. The theoretical framework in this field is based on the general idea that relative brain size evolves through a balance between the positive fitness effects of increased cognitive ability and the prohibiting effects of the energetic costs of developing and maintaining a larger brain (e.g. Soemmerring, 1785; Darwin, 1871; Jerison, 1973; Aiello & Wheeler, 1995; Striedter, 2005; Chittka & Niven, 2009; Navarrete *et al*., 2011; Kotrschal *et al*., 2013a).

Empirical evidence for positive effects of increased brain size stems mainly from comparative studies where larger brains have been associated with higher frequencies of cognitively demanding behaviours such as parental care, tool use and social behaviour (Gittleman, 1994; Dunbar, 1998; Gonzalez‐Voyer *et al*., 2009; Brown, 2012; Kotrschal *et al*., 2013a). Comparative analyses have shown that larger brains can be associated with ecological variables such as novel or challenging environments (Sol *et al*., 2007; Maklakov *et al*., 2011; Snell‐Rood & Wick, 2013; Husby & Husby, 2014). Recently, experimental evidence for a causal link between brain size and cognitive ability was provided based on artificial selection on brain size in the guppy (*Poecilia reticulata*). In a test of numerical learning ability, large‐brained guppy females outperformed their small‐brained peers (Kotrschal *et al*., 2013a,b). Moreover, using the same brain size selection lines, it was also demonstrated that large‐brained males were faster at learning to find a potential mate in a spatial maze (Kotrschal *et al*., 2014).

Although a larger brain offers cognitive advantages, it is also a highly costly organ to develop and maintain (Aiello & Wheeler, 1995). For instance, the human brain constitutes roughly 2% of the total body mass but utilizes over 20% of the total energy budget (Aiello & Wheeler, 1995). The ‘expensive tissue’ hypothesis (Aiello & Wheeler, 1995) assumes that individuals can invest only a given amount of energy into organ growth and predicts that due to the energetic costs involved in developing a larger brain, trade‐offs will occur between brain size and other expensive tissues such as the gut. Indeed, several comparative studies have demonstrated negative associations between brain size and gut size (Aiello & Wheeler, 1995; Aiello *et al*., 2001; Tsuboi *et al*., 2014). This hypothesis assumes that any reduction in gut size is coupled to a switch in diet to higher quality or more easily digestible food, and the hypothesis has recently been extended to explain negative associations found between brain size and other energetically costly organs such as fat storage (Navarrete *et al*., 2011; but see Speijer, 2012), muscle tissue (Isler & van Schaik, 2006) or reproductive effort (Isler & van Schaik, 2009). An experimental study in guppies further showed that individuals artificially selected for larger brains exhibited reduced gut size and also reduced fecundity compared to individuals selected for smaller brains (Kotrschal *et al*., 2013a).

Primary sexual traits (sex organs or genitals that are directly necessary for reproduction) and secondary sexual traits (sex‐specific traits or ornaments that are indirectly necessary for reproduction) are remarkably variable in both animals and plants and are fundamentally important for reproductive success (Willson, 1979; Andersson, 1994; Arnqvist & Rowe, 2005). Primary and secondary sexual traits are also highly costly to develop and maintain (Zahavi, 1975; Kodric‐Brown & Brown, 1984; Andersson, 1994) and frequently subject to trade‐offs (Emlen, 2001). Given the costly nature of the brain and of sexual traits, it is intuitive to extend the costly tissue hypothesis to sexual traits predicting a trade‐off between brain size and the development of sexual traits. Interestingly, very few studies have tested for negative associations between brain size and sexual traits and the results remain inconclusive. We are only aware of one such demonstration, a phylogenetic comparative analysis that found a negative association between testis size and brain size across bat species (Pitnick *et al*., 2006). This data set was later extended to more mammal groups and reanalysed in another study that found no overall negative relationship between brain size and sexual traits (Lemaitre *et al*., 2009). Given the lack of data on the potential trade‐off between brain size and sexual traits, additional studies on this topic are essential to broaden our understanding of the potential constraints and limitations that affect brain evolution.

An alternative hypothesis may suggest positive associations between brain size and sexually selected traits via sexual selection on cognitive ability and therefore brain size (Boogert *et al*., 2011). One rationale behind this idea is that individuals with larger brains are better at foraging and exploiting food resources, obtain overall better condition and therefore develop more elaborate sexual traits. Condition can be broadly defined as the total pool of resources available for allocation to different traits (Rowe and Houle 1996). Male traits are often condition dependent (Kodric‐Brown & Brown, 1984; Johnstone, 1995; Bonduriansky, 2007), and examples of a direct association between foraging behaviour and the expression level of sexually selected traits can be found in the house finch (*Carpodacus mexicanus*), where individuals with a higher carotenoid pigment concentration in their gut showed brighter plumage coloration (Badyaev & Hill, 2002). Similarly, wild‐caught male guppies exhibiting spots with higher colour saturation were better at finding algae in a maze (Karino *et al*., 2005). Given the contrast between the two existing theories concerning the association between brain size and sexually selected traits and the paucity of experimental data on the subject, additional empirical data are imperative.

**Figure 1:**
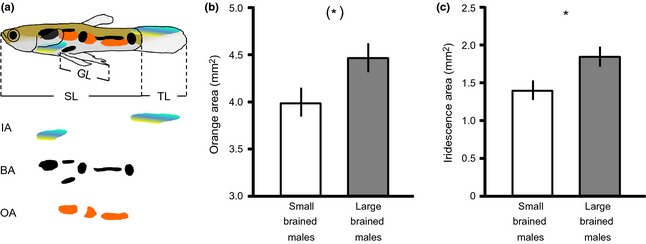
Coloration in male guppies selected for large and small relative brain size. Measured components (a) included standard length (SL), tail length (TL), gonopodium length (GL), iridescent area (IA), black area (BA) and orange area (OA). Large‐brained males show 11.8% larger orange spots (b) and a 31.8% greater area of iridescence (c). Asterisks indicate significant differences ((*)*P* < 0.1; *P < 0.05). Shown are the mean estimated marginal means (±SE) of general linear mixed models (GLMMs) controlling for body size and replicate population (see main text).

Here, we test the direction of the association between relative brain size and several male primary and secondary sexually selected traits using recently developed artificial selection lines differing in brain size in the guppy. These lines have been selected for either large or small relative brain size over three generations leading to a divergence of 9–14% in relative brain size, and significant differences in both female and male cognitive ability between the lines (Kotrschal *et al*., 2013a,b, 2014). To investigate the relationship between brain size and male sexual traits, we compare both primary male traits (gonopodium length, testis size and sperm number) and secondary male traits (carotenoid spot area, carotenoid spot intensity, melanin spot coloration and tail fin length; see Fig. 1a) between the smaller‐ and larger‐brained lines. The investigated primary and secondary male traits are known to play an important role for male reproductive success in the guppy (sperm number, Boschetto *et al*., 2011; gonopodium length, e.g. Jennions & Kelly, 2002; Kelly *et al*., 2000; various aspects of carotenoid and melanin coloration, e.g. Houde, 1997 and references therein; tail length, Bischoff *et al*., 1985) and are therefore highly suitable for the targeted comparisons with artificially selected variation in relative brain size. In addition, coloration in the guppy is known to be condition dependent, which supports the idea that these traits are costly to produce (Houde, 1997 and references therein, Devigili *et al*., 2013; Rahman *et al*., 2013). Given that condition may influence both sexual trait formation and neural investment (Catchpole, 1996; Nowicki *et al*., 1998; Georgieff, 2007), we further compared body condition in individuals kept in breeding pairs and in larger same‐sex groups.

If the expensive tissue hypothesis adequately describes the relationship between brain size and the size of primary and secondary sexual traits, we predict lower expression of sexual traits in the larger‐brained males. Under the alternative ‘positive association hypothesis’, we predict greater expression of sexual traits in the larger‐brained males. If condition mediates the relationship between brain size and sexual traits, larger‐brained males should show higher body condition.

## Materials and methods

### Directional selection on brain weight

We examined the relationship between brain size and sexual traits in laboratory lines of Trinidadian guppies that were artificially selected for large or small relative brain size (Kotrschal *et al*., 2012, 2013a). Briefly, these selection lines were generated using a standard bidirectional artificial selection design that consisted of two replicated treatments (three upselected lines and three downselected lines). As brain size can only be quantified after dissection, we allowed pairs to breed at least two clutches first and then killed the parents for brain quantification and used the offspring from parents with large or small relative brain size as parents for the next generation. More specifically, to select for relative brain size (controlled for body size), we selected on the residuals from the regression of brain size (weight) on body size (length) of both parents. We started with three times 75 pairs (75 pairs per replicate) to create the first three ‘upselected’ and ‘downselected’ lines (six lines in total). We summed up the male and female residuals for each pair and used offspring from the top and bottom 25% of these ‘parental residuals’ to form the next‐generation parental groups. We then used the offspring of the 30 pairs with the largest residual sums for upselection and the 30 pairs with the smallest residual sums for downselection for each following generation. To avoid inbreeding, full‐siblings were never mated. See Kotrschal *et al*. (2013a) for full details on the selection experiment. The selection lines differ in relative brain size by 9% in F2 (Kotrschal *et al*., 2013a) and up to 14% in F3 (Kotrschal *et al*., 2014), and body size does not differ between the lines (Kotrschal *et al*., 2013a, 2014). All fish were removed from their parental tanks after birth, separated by sex at the first onset of sexual maturation and then kept in single‐sex groups with a maximum density of five individuals in 3‐L tanks containing 2 cm of gravel with continuously aerated water. We allowed for visual contact between the tanks. The laboratory was maintained at 26 °C with a 12:12 light:dark schedule. Fish were fed a diet of flake food and freshly hatched brine shrimp 6 days per week. All measurements were taken blindly as only running‐numbers identified tanks. We used several different groups of fully grown and mature F3 male guppies for our assays. The groups were balanced over the three replicates and the two brain size selection regimes. We used 180 individuals to determine gonopodium size, testis size, carotenoid spot area, melanin spot area, iridescent pattern area, tail length, and body condition in individuals kept in pairs; 180 additional individuals for spectrophotometric measurements; 60 individuals for sperm quantification; and finally 30 individuals for quantification of condition in individuals kept in larger groups.

### Male structural traits

To explore the relationship between brain size and male structural traits, we quantified gonopodium size, testis size, carotenoid, melanin spot area, area of iridescence and tail length. To do this, fish (*n* = 180) were first euthanized with an overdose of benzocaine and measured for standard length (SL) (from the tip of the snout to the end of the caudal peduncle, Fig. 1a) to the nearest 0.01 mm using digital callipers.

After measurements, each fish was submerged in a small Petri dish filled with water and lateral pictures of the left body side were taken through a dissecting microscope (Leica MZFLIII and FiRECAM v. 3.1, Leica Microsystems, Heerburg, Germany). We used ImageJ (1.43u NIH, US National Institutes of Health, MD, USA) to determine gonopodium length (measured from the base of the gonopodium to the tip of the last fin ray), tail length (measured from the end of the caudal peduncle to the tip of the middle tail ray), and size of carotenoid (orange) spot, melanin (black) spot and iridescent areas (Fig. 1a). To quantify colour spot areas, manual outlines of all orange, black and iridescent spots were made and the values for cumulative numbers of orange, black and iridescent area were calculated (Fig. 1a). The males were subsequently placed in 5% buffered formalin for fixation. After fixation (90.1 ± 0.1 days in formalin), the testes were removed under a stereomicroscope and weighed to the nearest 0.001 mg.

To determine how brain size selection influences carotenoid colour intensity, spectrophotometric measurements were performed. To do this, fish (*n* = 180) were anaesthetized with benzocaine in the holding water (2.85 m) and an Ava‐Spec 2048FT‐SPU (Avantes Inc., Apeldoorn, Netherlands) spectrophotometer was used to take five measurements of transmittance values of the flank orange spot from 300 to 700 nm. To maximize long‐wave (500–700 nm) transmittance, the angle of the detector was varied for each measurement within a range of 10°. The five measurements were then averaged to produce one transmittance spectrum per individual, which consisted of 401 values per individual (one per wavelength). Hue (wavelength of maximal transmittance), saturation (difference between maximal and minimal transmittance) and brightness (sum of transmittance of all wavelengths) were determined for every individual (Hill & McGraw, 2006) in the single orange peak (500–700 nm). Measurements were grouped into 10‐nm bins from which the median value was used to represent the respective bin (Hill & McGraw, 2006).

For determination of sperm number, an important trait for post‐copulatory sexual selection (Parker, 1993), fish (*n* = 60) were anaesthetized by submersion in ice slurry for 5‐s and then placed left side up on a black plastic slide with a drop (5 μL) of saline solution (0.9% sodium chloride). SL was measured and sperm reserves (organized in bundles) were extracted under a dissecting microscope by gently brushing a blunt probe along the male's anterior abdomen, with the gonopodium swung forward. All sperm bundles were carefully sucked up in small amounts of saline solution using a 10‐μL micropipette and stored in droplets on a plastic slide for counting. All experimental fish were successfully revived in warm aerated water. After quantification of all visible spermatozeugmata, all bundles were collected in 0.5‐mL Eppendorf tubes containing a total of 30 μL saline solution. To obtain sperm cell counts, the homogenized saline/sperm solution was sucked up and sperm bundles were released into a 10‐μL micropipette 40 times, followed by examination of 5 μL of the mixture using the Integrated Semen Analysis System software (ISAS; Proiser, Valencia, Spain) at 10× magnification. We examined a minimum of ten separate fields for each fish, using at least two different aliquots of sperm mixture from each ejaculate. Total sperm number was determined by multiplying the mean number of sperm per field in the ISAS software for each ejaculate by the sample's dilution factor and initial volume. For this analysis, we closely followed the protocol by Matthews *et al*. (1997), a method characterized by a high repeatability (Zajitschek *et al*., 2009).

### Male condition

We quantified condition both in the pair‐housed males that were used for the quantification of male structural traits and in males kept in same‐sex larger groups. We used body length and body weight (measured to the nearest 0.1 mg) data to quantify the Fulton's index of body condition, a highly suitable indicator of body fat content in small fish (Kotrschal *et al*., 2011). The Fulton index *K* was calculated as *K = M/SL* ^*3*^ * 100 g mm^−3^, where *M* is the fish's body mass [g] and *SL* is its SL [mm] (Bolger & Connolly, 1989). To determine body condition of large‐ and small‐brained fish in nonreproducing fish kept in larger groups, we removed adult fish from stock tanks (where they had been kept in large same‐sex tanks) and placed 18 adult males each (separated by brain size and replicate) in six 45‐L tanks. Those tanks were equipped with a layer of gravel, some java moss and a biological filter. Once per day we fed the fish in those six tanks (large‐/small‐brained and three replicates) an *ad libitum* ration of live brine shrimp and flake food for 4 weeks and then determined body size (SL using a measuring board to the nearest 0.5 mm) and body weight (padded dry animals to the nearest 1 mg) of five randomly chosen fish per group (*N* = 30) in the morning before fish were fed. We used these measures of body size and weight to determine body condition analogously to the pair‐housed fish.

### Statistical analyses

To check whether the fish used for male sexual traits assay were of equal age and size, we used two general linear mixed models (GLMMs) with age (days) and size (mm) as dependent variables, brain size as fixed and replicate nested in brain size selection regime as random effects. To then test for differences between large‐ and small‐brained males in sexually selected traits, we built separate analogous GLMMs. We included the trait of interest as dependent variable, brain size as fixed and replicate nested in brain size selection regime as random effect. The size of structural traits is usually highly correlated with body size; we therefore used body size as covariate where necessary. We performed a stepwise model selection based on Akaike's Information Criterion (AIC), only varying the fixed effects structure (Zuur *et al*., 2009), using a full‐interaction model as starting model and disregarding interactions when nonsignificant. We used an analogous GLMM to analyse body condition of fish kept in group tanks with body condition as dependent variable, brain size selection regime as fixed and replicate nested in brain size selection regime as random factor. Sperm number was square‐root‐transformed to meet normality criteria. To obtain an estimate of the orange spectra, we performed a principle component analysis on 10‐nm bins and used the first component (explaining 77.9% of the variance) as dependent variable in another GLMM built analogously to the ones described above. Because we had clear predictions based on previous studies for each of the measured traits (Bischoff *et al*., 1985; Houde, 1997; Kelly *et al*., 2000; Magurran, 2005; Shohet & Watt, 2009; Boschetto *et al*., 2011), we kept *α *= 0.05 and did not employ multiple testing corrections (Nakagawa, 2004). The analyses were carried out in SPSS 19.0 (SPSS Inc, Chicago, IL, USA) and in the R statistical environment (R Core Team, 2014).

## Results

The males used for the assays of male sexual ornaments were all of similar age and size (GLMM_age_: *F*_1,7_ = 0.83, *P* = 0.458, age [days, mean ± SE]: small‐brained: 132.3 ± 1.2; large‐brained: 135.5 ± 1.3; and GLMM_size_: *F*_1,7_ = 0.33, *P* = 0.624, SL [mm, mean ± SE]: small‐brained: 15.99 ± 0.17; large‐brained: 15.84 ± 0.17).

**Figure 2:**
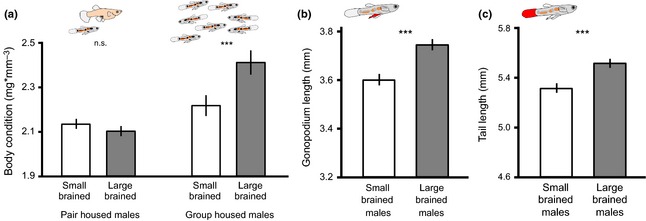
Difference in morphological traits of male guppies selected for large and small brain size. Body condition only differs in groups‐housed males (a). The gonopodium is the male intermittent organ used for both sneak and solicited copulations and is 4.0% longer in large‐brained males (b). The tail is similarly 3.8% longer (c). Asterisks indicate significant differences (***P < 0.001). Shown are the mean estimated marginal means (±SE) of general linear mixed models (GLMMs) controlling for body size (b and c) and replicate population (a, b and c; see main text).

Males from the large‐brained lines had longer gonopodia (Table 1; Fig. 2a) and longer tail fins (Table 1; Fig. 2b) than males from the small‐brained lines.

**Table 1:** Differences in primary and secondary male sexual traits from guppies artificially selected for large and small relative brain size. Statistically significant results (*P* < 0.05) are highlighted in bold

	Gonopodium length			Tail length			Condition index *K*			Testis mass			Sperm bundle number			Spermatozoon number		
	d.f.	χ^2^	*P*	d.f.	χ^2^	*P*	d.f.	χ^2^	*P*	d.f.	χ^2^	*P*	d.f.	χ^2^	*P*	d.f.	χ^2^	*P*
Brain size selection	1/7	16.76	**<0.001**	1/7	12.45	**<0.001**	1/6	0.38	0.540	1/7	0.44	0.507	1/7	0.04	0.834	1/7	0.31	0.581
Body size	1/7	77.62	**<0.001**	1/7	27.19	**<0.001**	–	–	–	1/7	36.90	**<0.001**	1/7	7.30	**0.007**	1/7	0.93	0.336

The area of black melanin spots did not differ between large‐ and small‐brained males (Table 2), but large‐brained males showed a trend towards a greater orange spot area (Table 2; Fig. 1b) and a significantly greater area of iridescence (Table 2; Fig. 1c). Neither the analysis of hue, saturation, brightness, nor the principle component analysis of those spectrophotometric measures revealed any significant differences in qualitative aspects of the orange coloration (Table 3). Similarly, neither testis size, total sperm number, nor the number of sperm bundles differed between the groups (Table 1).

**Table 2:** Differences in guppy male coloration from individuals artificially selected for large and small relative brain size. Statistically significant results (*P* < 0.05) are highlighted in bold

	Melanin body area			Orange body area			Iridescence body area		
	d.f.	χ^2^	*P*	d.f.	χ^2^	*P*	d.f.	χ^2^	*P*
Brain size selection	1/7	0.78	0.377	1/7	3.36	0.067	1/7	5.60	**0.018**
Body size	1/7	9.13	**0.003**	1/7	5.11	**0.024**	1/7	0.64	0.424

**Table 3:** Aspects of guppy male carotenoid coloration measured from orange spots of male guppies artificially selected for large and small relative brain size

	Orange hue			Orange saturation			Orange brightness			Orange spectrum PCA		
	d.f.	χ^2^	*P*	d.f.	χ^2^	*P*	d.f.	χ^2^	*P*	d.f.	χ^2^	*P*
Brain size selection	1/6	1.28	0.259	1/6	0.09	0.765	1/6	1.48	0.224	1/6	1.21	0.271

Although condition did not differ in pair‐housed males (Table 1; Fig. 2a), large‐brained males that were kept in larger groups had a higher body condition than small‐brained males kept under the same conditions (GLMM: brain size: *F*_1,2_ = 7.63, *P* = 0.010; Fig. 2a).

## Discussion

An important aspect of brain size evolution is the potential trade‐off with other costly features including vital organs as well as costly sexually selected traits (Aiello & Wheeler, 1995; Isler & van Schaik, 2006). Indeed, we found support for such a trade‐off in a previous study where gut mass and fecundity was lower in large‐brained fish (Kotrschal *et al*., 2013a). In our current study however, we found no evidence for negative associations between brain size and male sexual traits, but in fact a higher expression of several male sexual traits in the larger‐brained males. More specifically, we found gonopodium length, orange spot area, iridescent spot area and body condition in single‐sex groups to be positively associated with increased brain size, whereas post‐copulatory sexual traits did not show any significant association with brain size. Several potential explanations for these patterns exist, which we discuss in more detail here below.

The first possible explanation for the lack of negative effects of an evolutionary increase in brain size on sexual traits is that brain size and sexual traits are not traded off against each other. It is notoriously difficult to provide indisputable demonstrations of the existence (or the nonexistence) of trade‐offs (Agrawal *et al*., 2010). However, using artificial selection on one trait and measuring the evolutionary response in other traits is considered to be the most powerful approach to investigating trade‐offs (Bell & Koufopanou, 1986; Conner, 2003; Fry, 2003; Agrawal *et al*., 2010). Hence, we argue that if strong trade‐offs between brain size and male sexual traits existed, we should have detected them in our artificial selection lines. So far, the traits that have been found to be negatively associated with brain size include gut size (Aiello & Wheeler, 1995; Isler & van Schaik, 2009; Kotrschal *et al*., 2013a; Tsuboi *et al*., 2014), muscle tissue (Isler & van Schaik, 2012), fat deposits (Navarrete *et al*., 2011; but see Speijer, 2012), brood size (Isler & van Schaik, 2006; Kotrschal *et al*., 2013a) and testis size (Pitnick *et al*., 2006; but see Lemaitre *et al*., 2009). In the light of this broad array of traits that are negatively associated with brain size, it is interesting that demonstrations of negative associations between the brain and sexual traits are so rare, especially considering the high energetic investment that sexual traits normally require (e.g. Andersson, 1994).

If our pattern of no negative correlation between brain size and sexual traits truly indicates lack of a trade‐off, we speculate that brain size and sexual traits may have temporally different developmental patterns and are therefore not in direct competition over resources. Guppies are live‐bearers, and in vertebrates with internal fertilization, brain development generally (human brain development forms an exception to this general rule, Bogin, 1999) occurs early during ontogenesis, that is prior to birth. In contrast to the early developmental pattern of the vertebrate brain, primary and secondary sexual traits show their main development later in life in most vertebrates (Andersson, 1994; Tam *et al*., 2003). We have previously demonstrated that the brain size differences in the artificial selection lines are evident already at birth (Kotrschal *et al*., 2013a), whereas the development of both primary and secondary male sexual traits in the guppy is known to occur much later in ontogenesis. Colour patterns, the gonopodium and sexual behaviours typically start developing at 3 months after birth (Evans *et al*., 2002; A. Kotrschal, personal observations). Therefore, separate energy budgets for the development of the brain and the male sexual traits could explain the lack of support for trade‐offs in this study.

Another explanation for the lack of negative associations between brain size and sexual traits is that selection on brain size has affected one or more genetic factors that underlie similar directional changes in both brain size and sexual traits. Males from the larger‐brained selection lines had longer gonopodia, longer tail fins and more iridescent coloration than males from the smaller‐brained lines. We also detected a nonsignificant trend (*P* = 0.067) towards more orange coloration in the larger‐brained males. If the same genetic architecture, through pleiotropy or genetic linkage (Jiang & Zeng, 1995), underlies phenotypic changes in brain size and male sexual traits, it could explain the observed pattern. Available examples of how sexual traits may be positively associated with other traits through pleiotropy or genetic linkage include covariation between male courtship behaviour and several life history traits, physiological processes and even brain size in *Drosophila* (reviewed in Fitzpatrick, 2004), and covariation between male sexual traits and body size in the guppy (Postma *et al*., 2011). A recent experimental study also showed that sexual selection is associated with improved cognitive performance in *Drosophila* males (Hollis & Kawecki, 2014). Hence, it is possible that these genetic mechanisms also drive the link between brain size and male sexual traits in the guppy.

A potential additional extension of this explanation is that artificial selection on brain size is affecting another trait (or set of traits) that in turn is positively correlated with both brain size and male sexual traits (Falconer & Mackay, 1996). Body condition is a potential candidate for such a ‘third variable’ that could be related to both brain size and the expression of male sexual traits. The brain is highly plastic in response to body condition as demonstrated for instance in birds (Catchpole, 1996; Nowicki *et al*., 1998; but see Gil *et al*., 2006) and in humans, where brain development is strongly dependent on nutrient status (e.g. Georgieff, 2007). Moreover, it was recently suggested that individuals with larger brains and higher cognitive abilities might be better foragers, leading to higher condition in individuals with better cognitive skills (Boogert *et al*., 2011). Similarly, male sexual traits are known to be condition dependent (Johnstone, 1995; Bonduriansky, 2007), which has for example been demonstrated for the orange coloration in the guppy (Devigili *et al*., 2013; Rahman *et al*., 2013). It is therefore possible that our artificial selection for increased and decreased relative brain size has also affected body condition in the selection lines. This idea is supported by the fact that although we did not find any effect of brain size on condition in males kept in pairs, we did find higher body condition in the large‐brained males as compared to the small‐brained males after they had been kept in larger single‐sex groups.

One explanation for this difference between pair‐ and group‐housed animals is the previously demonstrated higher stress tolerance of the larger‐brained animals, compared to the smaller‐brained animals (Kotrschal *et al*., 2014). Stress can have strong negative effects on condition (e.g. Barton & Iwama, 1991), and the effect on condition might be caused by larger‐brained males being better able to adjust to stress caused by male–male aggression than smaller‐brained males. Another explanation for this pattern could be that enhanced foraging abilities in larger‐brained males are only obvious in larger groups with higher competition for resources. We note, however, that we used an *ad libitum* feeding protocol in all set‐ups, so food restrictions are unlikely to have occurred. In any case, more studies are necessary to reveal the exact mechanism behind the association between brain size and body condition.

What is the ecological relevance of the greater expression of primary and secondary sexual traits in the larger‐brained male guppies? As mentioned previously, the area of orange spots tended to be greater and the area of the iridescent patterns was greater in larger‐brained males compared to smaller‐brained males, whereas the black spot area and the qualitative aspects of the orange colour spots did not differ between the two groups. In several dichotomous mate choice experiments designed to test for colour preference, female guppies have been shown to consistently choose males with larger colour areas, whereas black is rarely a preferred trait (see table p. 48 in Houde, 1997). It is therefore likely that the colour pattern of larger‐brained males would be more attractive to female guppies than those of smaller‐brained males. In addition, we found longer gonopodia and longer tail fins in males selected for large brains and these traits have also been shown to positively influence mate choice in female guppies (longer gonopodia, Brooks & Caithness, 1995; longer tails, Bischoff *et al*., 1985). Multiple traits important in precopulatory mate choice are therefore associated with a larger brain. At the same time, the longer gonopodia in the larger‐brained males may also render them an additional selective advantage as gonopodium length facilitates fertilization success during forced copulations (Evans *et al*., 2011; but see Gasparini *et al*., 2011). Together, these patterns are consistent with the idea that brain size and cognitive ability could coevolve with both sexual traits and behavioural traits that are important during mating (Jacobs, 1996; Miller, 2000; Boogert *et al*., 2011; Kotrschal *et al*., 2012, 2014). Future studies will aim at investigating the possible differences in mating success between males and females with varying brain size to elucidate the link between brain size, cognition, attractiveness and mating success. But are the larger‐brained males developing towards ‘Darwinian demons’ (Leimar, 2001) due to their superior cognitive ability and likely greater mating success? This is unlikely, because larger‐brained guppies show a decreased fecundity (Kotrschal *et al*., 2013a) and their smaller guts (Kotrschal *et al*., 2013a) may render them ill‐adapted for low‐food environments. Again, future studies will focus on disentangling under what exact conditions a larger brain may (or may not) convey fitness benefits.

As mentioned above, although we detected the described positive correlation between male brain size and precopulatory sexual traits, we did not find any association between male brain size and post‐copulatory traits. Neither testes mass, sperm number, nor the number of sperm bundles differed between large‐ and small‐brained males. These results suggest that traits used in precopulatory mate choice are positively associated with brain size, whereas traits used in post‐copulatory sexual selection do not show any link with brain size. This is somewhat surprising as also post‐copulatory sexual traits have been found to be condition dependent in several taxa (e.g. Schulte‐Hostedde *et al*., 2005; Burness *et al*., 2008) including the guppy (Simmons & Kotiaho, 2002; Rahman *et al*., 2013). Therefore, we speculate that the divergent results between pre‐ and post‐copulatory sexual traits in relation to brain size may be due to differences in their genetic architecture. Specifically, the precopulatory traits associated with male attractiveness may be more closely linked with brain size than the post‐copulatory traits studied here. Forthcoming studies will focus on unravelling the genetic link between brain size and pre‐ and post‐copulatory male sexual traits in these selection lines.

In conclusion, we found no support for the expensive tissue hypothesis, which predicts a trade‐off between brain size and male sexual traits. Instead, we found support for positive effects of selection for brain size on several male sexual traits that are important for female mate choice. Although we cannot yet address the exact mechanism yielding the positive association between brain size and male sexual traits, we speculate that it may be mediated through positive genetic correlations caused by pleiotropy, genetic linkage or a common genetic background for overall condition. Future studies of the association between brain size, cognition and sexual traits may thus form a fruitful avenue to identify potential factors affecting brain size variation in natural populations.

## References

[jeb12608-bib-0001] Agrawal, A.A. , Conner, J.K. & Rasmann, S. 2010 Tradeoffs and adaptive negative correlations in evolutionary ecology In: Evolution after Darwin: the First 150 Years (Bell M., Eanes W., Futuyma D. & Levinton J., eds), pp. 243–268. Sinauer Associates, Sunderland, MA.

[jeb12608-bib-0002] Aiello, L.C. & Wheeler, P. 1995 The expensive‐tissue hypothesis – the brain and the digestive system in human and primate evolution. Curr. Anthropol. 36: 199–221.

[jeb12608-bib-0003] Aiello, L.C. , Bates, N. & Joffe, T. 2001 In defense of the expensive tissue hypothesis In: Evolutionary Anatomy of the Primate Cerebral Cortex (Falk D. & Gibson K.R., eds), pp. 57–78. Cambridge University Press, Cambridge.

[jeb12608-bib-0004] Andersson, M. 1994 Sexual Selection. Princeton University Press, Princeton, New Jersey.

[jeb12608-bib-0005] Arnqvist, G. & Rowe, L. 2005 Sexual Conflict. Princeton University Press, Princeton.

[jeb12608-bib-0006] Badyaev, A.V. & Hill, G.E. 2002 Paternal care as a conditional strategy: distinct reproductive tactics associated with elaboration of plumage ornamentation in the house finch. Behav. Ecol. 13: 591–597.

[jeb12608-bib-0007] Barton, B.A. & Iwama, G.K. 1991 Physiological changes in fish from stress in aquaculture with emphasis on the response and effects of corticosteroids. Annu. Rev. Fish Dis. 1: 3–26.

[jeb12608-bib-0008] Bell, G. & Koufopanou, V. 1986 The cost of reproduction In: Oxford Surveys in Evolutionary Biology, Vol. 3 (Dawkins R. & Ridley M., eds), pp. 83–131. Oxford University Press, Oxford.

[jeb12608-bib-0009] Bischoff, R.J. , Gould, J.L. & Rubenstein, D.I. 1985 Tail size and female choice in the Guppy (Poecilia‐Reticulata). Behav. Ecol. Sociobiol. 17: 253–255.

[jeb12608-bib-0010] Bogin, B. 1999 Patterns of Human Growth. Cambridge University Press, Cambridge, UK.

[jeb12608-bib-0011] Bolger, T. & Connolly, P.L. 1989 The selection of suitable indexes for the measurement and analysis of fish condition. J. Fish Biol. 34: 171–182.

[jeb12608-bib-0012] Bonduriansky, R. 2007 The evolution of condition‐dependent sexual dimorphism. Am. Nat. 169: 9–19. 17206580 10.1086/510214

[jeb12608-bib-0013] Boogert, N.J. , Fawcett, T.W. & Lefebvre, L. 2011 Mate choice for cognitive traits: a review of the evidence in nonhuman vertebrates. Behav. Ecol. 22: 447–459.

[jeb12608-bib-0014] Boschetto, C. , Gasparini, C. & Pilastro, A. 2011 Sperm number and velocity affect sperm competition success in the guppy (*Poecilia reticulata*). Behav. Ecol. Sociobiol. 65: 813–821.

[jeb12608-bib-0015] Brooks, R. & Caithness, N. 1995 Female choice in a feral guppy population – are there multiple cues. Anim. Behav. 50: 301–307.

[jeb12608-bib-0016] Brown, C. 2012 Tool use in fishes. Fish Fish. 13: 105–115.

[jeb12608-bib-0017] Burness, G. , Schulte‐Hostedde, A.I. & Montgomerie, R. 2008 Body condition influences sperm energetics in lake whitefish (*Coregonus clupeaformis*). Can. J. Fish. Aquat. Sci. 65: 615–620.

[jeb12608-bib-0018] Catchpole, C.K. 1996 Song and female choice: good genes and big brains? Trends Ecol. Evol. 11: 358–360. 21237878 10.1016/0169-5347(96)30042-6

[jeb12608-bib-0019] Chittka, L. & Niven, J. 2009 Are bigger brains better? Curr. Biol. 19: R995–R1008. 19922859 10.1016/j.cub.2009.08.023

[jeb12608-bib-0020] Conner, J.K. 2003 Artificial selection: a powerful tool for ecologists. Ecology 84: 1650–1660.

[jeb12608-bib-0021] Darwin, C. 1871 The Descent of Man and Selection in Relation to Sex. John Murray, London.

[jeb12608-bib-0022] Devigili, A. , Kelley, J.L. , Pilastro, A. & Evans, J.P. 2013 Expression of pre‐ and postcopulatory traits under different dietary conditions in guppies. Behav. Ecol. 24: 740–749.

[jeb12608-bib-0023] Dunbar, R.I.M. 1998 The social brain hypothesis. Evol. Anthropol. 6: 178–190.

[jeb12608-bib-0024] Emlen, D.J. 2001 Costs and the diversification of exaggerated animal structures. Science 291: 1534–1536. 11222856 10.1126/science.1056607

[jeb12608-bib-0025] Evans, J.P. , Pitcher, T.E. & Magurran, A.E. 2002 The ontogeny of courtship, colour and sperm production in male guppies. J. Fish Biol. 60: 495–498.

[jeb12608-bib-0026] Evans, J.P. , Gasparini, C. , Holwell, G.I. , Ramnarine, I.W. , Pitcher, T.E. & Pilastro, A. 2011 Intraspecific evidence from guppies for correlated patterns of male and female genital trait diversification. Proc. R. Soc. B Biol. Sci. 278: 2611–2620. 10.1098/rspb.2010.2453 PMC3136825 21270040

[jeb12608-bib-0027] Falconer, D.S. & Mackay, T.F.C. 1996 Introduction to Quantitative Genetics, 4th edn Longmany Green, Harlow, Essex, UK.

[jeb12608-bib-0028] Fitzpatrick, M.J. 2004 Pleiotropy and the genomic location of sexually selected genes. Am. Nat. 163: 800–808. 15266379 10.1086/386297

[jeb12608-bib-0029] Fry, J.D. 2003 Detecting ecological trade‐offs using selection experiments. Ecology 84: 1672–1678.

[jeb12608-bib-0030] Gasparini, C. , Pilastro, A. & Evans, J.P. 2011 Male genital morphology and its influence on female mating preferences and paternity success in Guppies. PLoS One 6: e22329. 21799825 10.1371/journal.pone.0022329 PMC3142123

[jeb12608-bib-0031] Georgieff, M.K. 2007 Nutrition and the developing brain: nutrient priorities and measurement. Am. J. Clin. Nutr. 85: 614S–620S. 17284765 10.1093/ajcn/85.2.614S

[jeb12608-bib-0032] Gil, D. , Naguib, M. , Riebel, K. , Rutstein, A. & Gahr, M. 2006 Early condition, song learning, and the volume of song brain nuclei in the zebra finch (*Taeniopygia guttata*). J. Neurobiol. 66: 1602–1612. 17058194 10.1002/neu.20312

[jeb12608-bib-0033] Gittleman, J.L. 1994 Female brain size and parental care in Carnivores. Proc. Natl. Acad. Sci. USA 91: 5495–5497. 8202515 10.1073/pnas.91.12.5495 PMC44022

[jeb12608-bib-0034] Gonzalez‐Voyer, A. , Winberg, S. & Kolm, N. 2009 Social fishes and single mothers: brain evolution in African cichlids. Proc. R. Soc. B Biol. Sci. 276: 161–167. 10.1098/rspb.2008.0979 PMC2614256 18796397

[jeb12608-bib-0035] Hill, G.E. & McGraw, K.J. 2006 Bird Coloration. Vol 1. Mechanisms and Measuremnts. Harvard University Press, Cambridge, MA.

[jeb12608-bib-0036] Hollis, B. & Kawecki, T.J. 2014 Male cognitive performance declines in the absence of sexual selection. Proc. R. Soc. B Biol. Sci. 281: 20132873. 10.1098/rspb.2013.2873 PMC3953837 24573848

[jeb12608-bib-0037] Houde, A. 1997 Sex, Color, and Mate Choice in Guppies. Princeton University Press, Princeton.

[jeb12608-bib-0038] Husby, A. & Husby, M. 2014 Interspecific analysis of vehicle avoidance behavior in birds. Behav. Ecol. 25: 504–508.

[jeb12608-bib-0039] Isler, K. & van Schaik, C. 2006 Costs of encephalization: the energy trade‐off hypothesis tested on birds. J. Hum. Evol. 51: 228–243. 16730368 10.1016/j.jhevol.2006.03.006

[jeb12608-bib-0040] Isler, K. & van Schaik, C.P. 2009 The expensive brain: a framework for explaining evolutionary changes in brain size. J. Hum. Evol. 57: 392–400. 19732937 10.1016/j.jhevol.2009.04.009

[jeb12608-bib-0041] Isler, K. & van Schaik, C.P. 2012 How our ancestors broke through the gray ceiling comparative evidence for cooperative breeding in early Homo. Curr. Anthropol. 53: S453–S465.

[jeb12608-bib-0042] Jacobs, L.F. 1996 Sexual selection and the brain. Trends Ecol. Evol. 11: A82–A86. 10.1016/0169-5347(96)81048-2 21237767

[jeb12608-bib-0043] Jennions, M.D. & Kelly, C.D. 2002 Geographical variation in male genitalia in *Brachyphaphis episcopi* (Poeciliidae): is it sexually or naturally selected? Oikos 97: 79–86.

[jeb12608-bib-0044] Jerison, H.J. 1973 Evolution of the Brain and Intelligence. Academic Press, New York.

[jeb12608-bib-0045] Jiang, C.J. & Zeng, Z.B. 1995 Multiple‐trait analysis of genetic‐mapping for quantitative trait loci. Genetics 140: 1111–1127. 7672582 10.1093/genetics/140.3.1111 PMC1206666

[jeb12608-bib-0046] Johnstone, R.A. 1995 Honest advertisement of multiple qualities using multiple signals. J. Theor. Biol. 177: 87–94.

[jeb12608-bib-0047] Karino, K. , Utagawa, T. & Shinjo, S. 2005 Heritability of the algal‐foraging ability: an indirect benefit of female mate preference for males’ carotenoid‐based coloration in the guppy, *Poecilia reticulata* . Behav. Ecol. Sociobiol. 59: 1–5.

[jeb12608-bib-0048] Kelly, C.D. , Godin, J.G.J. & Abdallah, G. 2000 Geographical variation in the male intromittent organ of the Trinidadian guppy (*Poecilia reticulata*). Can. J. Zool. 78: 1674–1680.

[jeb12608-bib-0049] Kodric‐Brown, A. & Brown, J.H. 1984 Truth in advertising – the kinds of traits favored by sexual selection. Am. Nat. 124: 309–323.

[jeb12608-bib-0050] Kotrschal, A. , Fischer, B. & Taborsky, B. 2011 An noninvasive method to determine fat content in small fish based on swim bladder size estimation. J. Exp. Zool. A Ecol. Genet. Physiol. 315A: 408–415. 21462352 10.1002/jez.686 PMC3358693

[jeb12608-bib-0051] Kotrschal, A. , Rogell, B. , Maklakov, A.A. & Kolm, N. 2012 Sex‐specific plasticity in brain morphology depends on social environment of the guppy, *Poecilia reticulata* . Behav. Ecol. Sociobiol. 66: 1485–1492.

[jeb12608-bib-0052] Kotrschal, A. , Rogell, B. , Bundsen, A. , Svensson, B. , Zajitschek, S. , Brannstrom, I. *et al* 2013a Artificial selection on relative brain size in the guppy reveals costs and benefits of evolving a larger brain. Curr. Biol. 23: 168–171. 23290552 10.1016/j.cub.2012.11.058 PMC3566478

[jeb12608-bib-0053] Kotrschal, A. , Rogell, B. , Bundsen, A. , Svensson, B. , Zajitschek, S. , Immler, S. *et al* 2013b The benefit of evolving a larger brain: big‐brained guppies perform better in a cognitive task. Anim. Behav. 86: e4–e6. 24109149 10.1016/j.anbehav.2013.07.011 PMC3791419

[jeb12608-bib-0054] Kotrschal, A. , Lievens, A.J.P. , Dahlbom, J. , Bundsen, A. , Semenova, S. , Sundvik, M. *et al* 2014 Artificial selection on relative brain size reveals a positive genetic correlation between brain size and proactive personality in the guppy. Evolution 68: 1139–1149. 24359469 10.1111/evo.12341 PMC4285157

[jeb12608-bib-0055] Kotrschal, A. , Corral Lopez, A. , Amcoff, M. & Kolm, N. 2014 A larger brain confers a benefit in a spatial mate search learning task in male guppies. Behav. Ecol. aru227. 10.1093/beheco/aru227 PMC4374130 25825587

[jeb12608-bib-0056] Leimar, O. 2001 Evolutionary change and Darwinian Demons. Selection 2: 65–72.

[jeb12608-bib-0057] Lemaitre, J.F. , Ramm, S.A. , Barton, R.A. & Stockley, P. 2009 Sperm competition and brain size evolution in mammals. J. Evol. Biol. 22: 2215–2221. 20069724 10.1111/j.1420-9101.2009.01837.x

[jeb12608-bib-0058] Magurran, A.E. 2005 Evolutionary Ecology: The Trinidadian Guppy. Oxford University Press, Oxford.

[jeb12608-bib-0059] Maklakov, A.A. , Immler, S. , Gonzalez‐Voyer, A. , Ronn, J. & Kolm, N. 2011 Brains and the city: big‐brained passerine birds succeed in urban environments. Biol. Lett. 7: 730–732. 21525053 10.1098/rsbl.2011.0341 PMC3169078

[jeb12608-bib-0060] Matthews, I.M. , Evans, J.P. & Magurran, A.E. 1997 Male display rate reveals ejaculate characteristics in the Trinidadian guppy *Poecilia reticulata* . Proc. R. Soc. B Biol. Sci. 264: 695–700.

[jeb12608-bib-0061] Miller, G. 2000 The Mating Mind. Vintage, London, UK.

[jeb12608-bib-0062] Nakagawa, S. 2004 A farewell to Bonferroni: the problems of low statistical power and publication bias. Behav. Ecol. 15: 1044–1045.

[jeb12608-bib-0063] Navarrete, A. , van Schaik, C.P. & Isler, K. 2011 Energetics and the evolution of human brain size. Nature 480: 91–93. 22080949 10.1038/nature10629

[jeb12608-bib-0064] Nowicki, S. , Peters, S. & Podos, J. 1998 Song learning, early nutrition and sexual selection in songbirds. Am. Zool. 38: 179–190.

[jeb12608-bib-0065] Parker, G.A. 1993 Sperm competition games – sperm size and sperm number under adult control. Proc. R. Soc. B Biol. Sci. 253: 245–254. 10.1098/rspb.1993.0110 8234363

[jeb12608-bib-0066] Pitnick, S. , Jones, K.E. & Wilkinson, G.S. 2006 Mating system and brain size in bats. Proc. R. Soc. B Biol. Sci. 273: 719–724. 10.1098/rspb.2005.3367 PMC1560082 16608692

[jeb12608-bib-0067] Postma, E. , Spyrou, N. , Rollins, L.A. & Brooks, R.C. 2011 Sex‐dependent selection differentially shapes genetic variation on and off the Guppy Y chromosome. Evolution 65: 2145–2156. 21790565 10.1111/j.1558-5646.2011.01314.x

[jeb12608-bib-0068] R Core Team 2014 R: A Language and Environment for Statistical Computing. R Foundation for Statistical Computing, Vienna, Austria.

[jeb12608-bib-0069] Rahman, M.M. , Kelley, J.L. & Evans, J.P. 2013 Condition‐dependent expression of pre‐ and postcopulatory sexual traits in guppies. Ecol. Evol. 3: 2197–2213. 23919162 10.1002/ece3.632 PMC3728957

[jeb12608-bib-0170] Rowe, L. & Houle, D. 1996 The lek paradox and the capture of genetic variance by condition dependent traits. Proc. R. Soc. B Biol. Sci. 263: 1415–1421.

[jeb12608-bib-0070] Schulte‐Hostedde, A.I. , Millar, J.S. & Hickling, G.J. 2005 Condition dependence of testis size in small mammals. Evol. Ecol. Res. 7: 143–149.

[jeb12608-bib-0071] Shohet, A.J. & Watt, P.J. 2009 Female guppies *Poecilia reticulata* prefer males that can learn fast. J. Fish Biol. 75: 1323–1330. 20738617 10.1111/j.1095-8649.2009.02366.x

[jeb12608-bib-0072] Simmons, L.W. & Kotiaho, J.S. 2002 Evolution of ejaculates: patterns of phenotypic and genotypic variation and condition dependence in sperm competition traits. Evolution 56: 1622–1631. 12353755 10.1111/j.0014-3820.2002.tb01474.x

[jeb12608-bib-0073] Snell‐Rood, E.C. & Wick, N. 2013 Anthropogenic environments exert variable selection on cranial capacity in mammals. Proc. R. Soc. B Biol. Sci. 280: 20131384. 10.1098/rspb.2013.1384 PMC3768300 23966638

[jeb12608-bib-0074] Soemmerring, S.T. 1785 über die körperliche verschiedenheit des Negers vom Europäer. Varrentrapp Sohn und Venner, Frankfurt am Main.

[jeb12608-bib-0075] Sol, D. , Szekely, T. , Liker, A. & Lefebvre, L. 2007 Big‐brained birds survive better in nature. Proc. R. Soc. B Biol. Sci. 274: 763–769. 10.1098/rspb.2006.3765 PMC2093983 17251112

[jeb12608-bib-0076] Speijer, D. 2012 Brains have a gut feeling about fat storage. BioEssays 34: 275–276. 22337576 10.1002/bies.201200002

[jeb12608-bib-0077] Striedter, G.F. 2005 Principles of Brain Evolution. Sinauer Associates, Sunderland.

[jeb12608-bib-0078] Tam, P.P.L. , Kanai‐Azuma, M. & Kanai, Y. 2003 Early endoderm development in vertebrates: lineage differentiation and morphogenetic function. Curr. Opin. Genet. Dev. 13: 393–400. 12888013 10.1016/s0959-437x(03)00085-6

[jeb12608-bib-0079] Tsuboi, M. , Husby, A. , Kotrschal, A. , Hayward, A. , Buechel, S. , Zidar, J. *et al* 2014 Comparative support for the expensive tissue hypothesis: big brains are correlated with smaller gut and greater parental investment in Lake Tanganyika cichlids. Evolution 69: 190–200. 25346264 10.1111/evo.12556 PMC4312921

[jeb12608-bib-0080] Willson, M.F. 1979 Sexual selection in plants. Am. Nat. 113: 777–790.

[jeb12608-bib-0081] Zahavi, A. 1975 Mate selection – selection for a handicap. J. Theor. Biol. 53: 205–214. 1195756 10.1016/0022-5193(75)90111-3

[jeb12608-bib-0082] Zajitschek, S.R.K. , Lindholm, A.K. , Evans, J.P. & Brooks, R.C. 2009 Experimental evidence that high levels of inbreeding depress sperm competitiveness. J. Evol. Biol. 22: 1338–1345. 19344380 10.1111/j.1420-9101.2009.01738.x

[jeb12608-bib-0083] Zuur, A.F. , Ieno, E.N. , Walker, N. , Saveliev, A.A. & Smith, G.M. 2009 Mixed Effects Models and Extensions in Ecology with R. Springer, Science & Business Media.

